# *In Vitro* and *In Vivo* Evaluation of the Antischistosomal Activity of Polygodial and 9-Deoxymuzigadial Isolated from *Drimys brasiliensis* Branches

**DOI:** 10.3390/molecules30020267

**Published:** 2025-01-11

**Authors:** Eric Umehara, Rayssa A. Cajas, Gabriel B. Conceição, Guilherme M. Antar, Adriano D. Andricopulo, Josué de Moraes, João Henrique G. Lago

**Affiliations:** 1 Centro de Ciências Naturais e Humanas, Universidade Federal do ABC, Santo André 09280-560, SP, Brazil; ericumehara@hotmail.com; 2 Núcleo de Pesquisas em Doenças Negligenciadas, Universidade Guarulhos, Guarulhos 07023-070, SP, Brazil; rayssacajas@gmail.com (R.A.C.); gabrielnpdn@gmail.com (G.B.C.); 3 Departamento de Ciências Agrárias e Biológicas, Universidade Federal do Espírito Santo, São Mateus 29932-540, ES, Brazil; guilherme.antar@gmail.com; 4 Laboratório de Química Medicinal e Computacional, Centro de Pesquisa e Inovação em Biodiversidade e Fármacos, Instituto de Física de São Carlos, Universidade de São Paulo, São Carlos 13563-120, SP, Brazil; aandrico@ifsc.usp.br; 5 Centro de Pesquisa e Inovação Especial em Ciências da Descoberta de Medicamentos (CEPIMED), Universidade de São Paulo, São Carlos 13563-120, SP, Brazil; 6 Núcleo de Pesquisas em Doenças Negligenciadas, Instituto Científico e Tecnológico, Universidade Brasil, São Paulo 08230-030, SP, Brazil

**Keywords:** antischistosomal activity, polygodial, 9-deoxymuzigadial, *Drimys brasiliensis*, schistosomiasis

## Abstract

In the present study, the hexane extract from branches of *Drimys brasiliensis* (Winteraceae) displayed potent activity against *Schistosoma mansoni* parasites (100% mortality of the worms at 200 μg/mL). Bioactivity-guided fractionation afforded, in addition to the previously reported bioactive sesquiterpene 3,6-epidioxy-bisabola-1,10-diene, two chemically related drimane sesquiterpenes—polygodial (**1**) and 9-deoxymuzigadial (**2**). The anti-*S. mansoni* effects for compounds **1** and **2** were determined in vitro, with compound **1** demonstrating significant potency (EC_50_ value of 10 μM for both male and female worms), while **2** was inactive. Cytotoxicity assays against Vero cells revealed no toxicity for either compound (CC_50_ > 200 μM). Additionally, an in silico analysis was conducted using the SwissADME platform for **1**, revealing that this natural sesquiterpene exhibited adherence to several ADME parameters and no PAINS violations. Finally, in vivo studies with *S. mansoni*-infected mice treated with compound **1** demonstrated a 44.0% reduction in worm burden, accompanied by decreases in egg production of 71.8% in feces and 69.5% in intestines. These findings highlight the potential of polygodial (**1**) as a promising prototype for schistosomiasis treatment.

## 1. Introduction

Neglected tropical diseases (NTDs), as defined by the World Health Organization (WHO), comprise a group of illnesses that predominantly impact populations in low- and middle-income countries, particularly in tropical and subtropical regions. These diseases often receive insufficient attention in terms of research and new treatment development, especially when compared to diseases more prevalent in high-income countries [1]. Among NTDs, schistosomiasis stands out as a significant public health challenge. This parasitic disease, caused by worms of the genus *Schistosoma*, can lead to severe damage to internal organs, particularly the liver and intestines, resulting in chronic morbidity and a diminished quality of life [2].

Aligned with Sustainable Development Goal (SDG) target 3, the elimination of NTDs, including schistosomiasis, has been prioritized globally. To this end, the WHO has outlined an ambitious roadmap to eliminate schistosomiasis as a public health problem by 2030 [3]. A key strategy in achieving this target is the development of novel antischistosomal agents [4]. Currently, schistosomiasis treatment relies almost exclusively on praziquantel, a therapy that has remained the cornerstone of treatment for decades. However, concerns regarding its long-term efficacy and the potential emergence of praziquantel-resistant Schistosoma strains highlight the pressing need to explore alternative therapeutic options [4,5].

*Drimys brasiliensis* Miers. (Winteraceae), commonly known in Brazil as “casca-d’anta”, has frequently been used in traditional medicine as an analgesic and anti-inflammatory agent [6]. Previous studies on *D. brasiliensis* described the isolation of several terpenoids including polygodial, 1β-(*p*-methoxy-cinnamoyl)-polygodial, 1β-coumaroyl-polygodial, and drimanial, which have been evaluated for their antifungal, anti-inflammatory, and cytotoxic effects [6,7,8,9]. Additionally, other studies have described the antiparasitic activity of polygodial and related compounds, particularly against the protozoans *Leishmania infantum*, *Plasmodium falciparum*, and *Trypanosoma cruzi* [10]. More recently, our group reported the anti-*S. mansoni* effects of 3,6-epidioxy-bisabola-1,10-diene, isolated from *D. brasiliensis* branches [11]. In continuation with this study, the effects of isolated sesquiterpenes polygodial (**1**) and 9-deoxymuzigadial (**2**) against *S. mansoni* were evaluated in vitro. Considering the potent antiparasitic activity of **1** and its adherence to several ADME parameters using in silico analysis (SwissADME platform), this compound was further assessed using an in vivo model.

## 2. Results

### 2.1. Chemical Characterization of Polygodial (***1***) and 9-Deoxymuzigadial (***2***)

The hexane extract from the branches of *D. brasiliensis* initially demonstrated 100% mortality against *Schistosoma mansoni* parasites and bioactivity-guided fractionation of the extract led to the isolation of, in addition to the previously reported bioactive sesquiterpene 3,6-epidioxy-bisabola-1,10-diene [11], compounds **1** and **2**.

**Figure 1:**
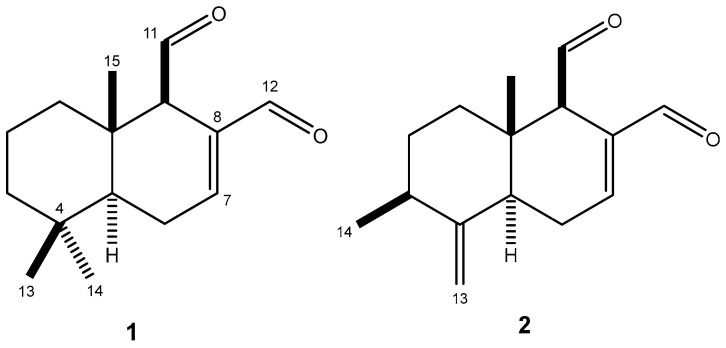
Structures of sesquiterpenes polygodial (**1**) and 9-deoxymuzigadial (**2**).

ESI-HRMS spectrum of **1** showed the [M + H]^+^ ion peak at *m*/*z* 235.1679, compatible with molecular formula C_15_H_22_O_2_. The ^1^H NMR spectrum displayed three singlets from methyl hydrogens at δ 0.96 (H-13), 0.92 (H-14), and 0.94 (H-15). Additionally, two characteristic aldehyde signals were observed at δ 9.53 (d, *J* = 4.4 Hz, H-11) and δ 9.45 (s, H-12), along with a double bond signal at δ 7.14 (t, *J* = 2.8 Hz, H-7), suggesting an α,β-unsaturated system. In the ^13^C NMR spectrum, 15 peaks of a sesquiterpene were observed, including two carbonyl carbon signals at δ 202.0 (C-11) and 193.2 (C-12), this being the last part of an α,β-unsaturated system due to the signals attributed to sp^2^ carbons at δ 154.3 (C-7) and 138.4 (C-8). Therefore, the structure of polygodial (**1**—Figure 1) was confirmed by a comparison of NMR data with those previously reported in the literature [9].

To compound **2**, ESI-HRMS spectrum showed the [M + H]^+^ ion peak at *m*/*z* 233.1557, compatible with molecular formula as C_15_H_20_O_2_. The ^1^H NMR of **2** showed similarities with that of **1** except for the presence of two singlets of an exocyclic double bond at δ 4.91 (H-13a) and 4.72 (H-13b), and one doublet at δ 1.08 (*J* = 6.5 Hz, H-14). Analysis of the ^13^C NMR spectrum confirmed the presence of one exocyclic double bond due to the peaks at δ 106.1 (C-13) and 151.4 (C-4) whereas those at δ 201.2 (C-11) and δ 193.4 (C-12) were assigned to the aldehyde carbons C-11 and C-12, respectively. Considering that the peaks assigned to C-7 and C-8 were observed, respectively, at δ 153.0 and δ 138.0, similarly to those of **1**, the differences between these sesquiterpenes are in the substituents at C-3 and C-4 positions. Therefore, the structure of 9-deoxymuzigadial (**2**—Figure 1) was confirmed by comparison of NMR data with those previously reported in the literature [12], this being the first occurrence of **2** in the genus *Drimys*.

### 2.2. In Vitro Evaluation of Polygodial (***1***) and 9-Deoxymuzigadial (***2***)

Following molecular characterization, compounds **1** and **2** were subjected to in vitro evaluation against *S. mansoni*. Adult worm pairs were analyzed separately as male and female parasites, given the known biological and physiological differences between sexes that can influence drug susceptibility. Cytotoxicity (CC_50_) was assessed using Vero mammalian cells, and the selectivity index (SI) was subsequently calculated. Praziquantel served as the positive control, while DMSO was used as the negative control (Table 1).

Polygodial (**1**) exhibited an EC_50_ value of approximately 10 μM for both male and female *S. mansoni* worms, demonstrating significant antischistosomal activity. In contrast, 9-deoxymuzigadial (**2**) showed no detectable activity against either sex of the parasite within the tested concentration range, classifying it as “not active”.

Cytotoxicity assays revealed no significant toxicity for either compound, with CC_50_ values exceeding 200 μM against Vero cells. The selective index (SI), calculated as the ratio of CC_50_ to EC_50_, was greater than 21 for polygodial (**1**), highlighting its favorable therapeutic profile. In comparison, praziquantel exhibited superior potency, with EC_50_ values of approximately 1 μM for *S. mansoni* and an SI greater than 150.

### 2.3. In Silico Evaluation of Polygodial (***1***)

**Figure 2:**
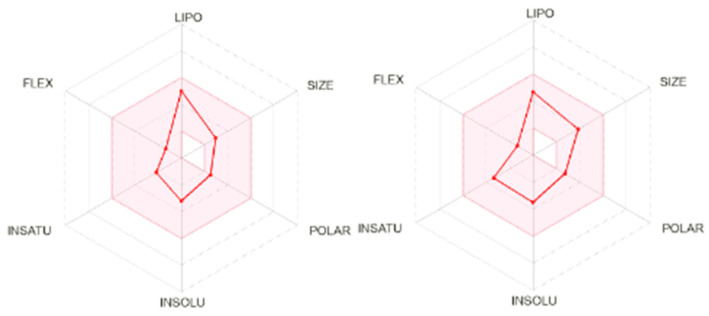
Bioavailability radar of polygodial (**1**, (**left**)) and positive control praziquantel (**right**).

To support the subsequent in vivo evaluation of polygodial (**1**), an in silico analysis was performed using the SwissADME platform to assess its pharmacokinetic and pharmacodynamic characteristics [13]. A key tool for interpreting these data is the bioavailability radar, which evaluates a drug’s absorption into the bloodstream and its biotransformation for excretion. As observed in Figure 2, polygodial (**1**) demonstrated good adherence to all evaluated parameters, similar to the drug praziquantel, used for the treatment of schistosomiasis.

Table 2 displays the in silico physicochemical properties and ADME parameters of polygodial (**1**) compared to praziquantel. As observed, tested compound **1** does not violate any of the drug-likeness rules set forth by Lipinski, Ghose, Veber, Egan, or Muegge, log P_o/w_ < 5, molecular weight (MW) < 400 Da, and a topological polar surface area (TPSA) < 140 Å. These values indicate high absorption potential and favorable oral bioavailability, making polygodial (**1**) a promising lead-like compound. Additionally, no PAINS (Pan Assay Interference Compounds) alerts were identified, further supporting its potential as a drug candidate.

### 2.4. In Vivo Evaluation

Building on the promising *in vitro* and in silico results, the in vivo efficacy of polygodial (**1**) was assessed in *S. mansoni*-infected mice. Animals were treated with a single oral dose of 400 mg/kg of either polygodial (**1**) or praziquantel, following a standardized protocol for evaluating antischistosomal drugs in murine models [14]. The results were compared to an infected, untreated control group.

**Figure 3:**
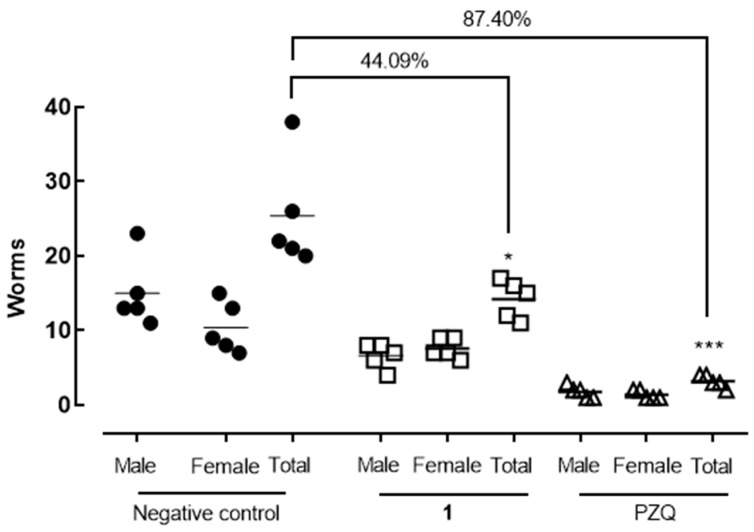
Effect of polygodial (**1**) and praziquantel (PZQ) on worm burden in *S. mansoni*-infected mice. Forty-two days post-infection, animals received a single oral dose of either the test compound (400 mg/kg) or the vehicle (control). At 56 days post-infection, animals were euthanized and dissected, and schistosomes were removed, sexed, and counted. Data points represent individual animals (*n* = 5 per group), with horizontal bars indicating median values. * *p* < 0.05; *** *p* < 0.001 compared to the infected vehicle-treated control group.

**Figure 4:**
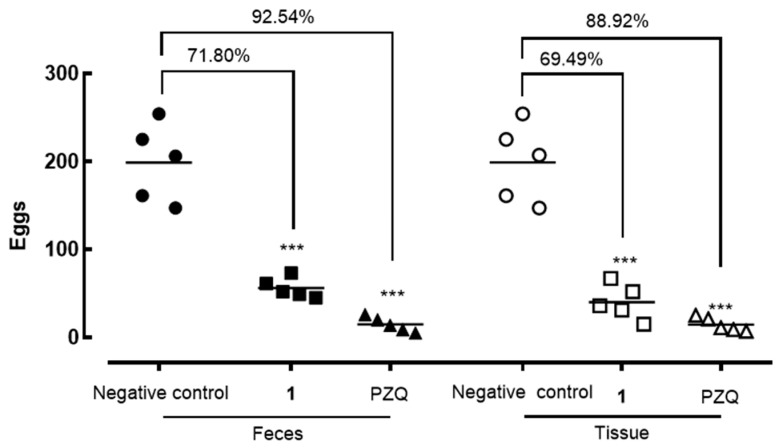
Effect of polygodial (**1**) and praziquantel (PZQ) on egg burden in *S. mansoni*-infected mice. Forty-two days post-infection, animals received a single oral dose of either the test compound (400 mg/kg) or the vehicle (control). At 56 days post-infection, mice were euthanized, and egg burden was assessed in both feces and intestinal tissue. Egg counts in intestinal tissue reflect the number of immature eggs. Data points represent individual animals (*n* = 5 per group), with horizontal bars indicating median values. *** *p* < 0.001 compared to the infected vehicle-treated control group.

Polygodial (**1**) treatment led to a significant reduction in worm burden, achieving a 44.09% (*p* < 0.05) decrease, while the reference drug praziquantel reduced worm burden by 87% (*p* < 0.001) (Figure 3). Additionally, compound **1** substantially reduced egg production, with decreases of 71.80% (*p* < 0.001) in fecal samples and 69.49% (*p* < 0.05) in intestinal tissues. In comparison, praziquantel reduced egg counts by 88–92% in both feces and intestinal tissues (Figure 4).

These findings highlight the potential of polygodial (**1**) to not only reduce the adult parasite load but also disrupt egg production, a critical factor in controlling disease transmission and pathology.

## 3. Discussion

Bioactivity-guided fractionation of hexane extract from branches of *D. brasiliensis* afforded two fractions—the first one composed exclusive of 3,6-epidioxy-bisabola-1,10-diene [11] and the second composed of sesquiterpenes **1** and **2**, which were purified by SiO_2_/AgNO_3_ column chromatography and characterized as polygodial and 9-deoxymuzigadial, respectively, by analysis of NMR and HR-ESIMS data (see Supplementary Materials).

The antischistosomal potential of polygodial (**1**) and 9-deoxymuzigadial (**2**), revealed significant in vitro activity for **1**, while **2** was inactive. In vitro, polygodial (**1**) exhibited notable activity against both male and female *S. mansoni* worms, with no cytotoxicity observed in mammalian Vero cells, yielding a high selectivity index (SI > 20). While praziquantel remains the gold standard for schistosomiasis treatment, with superior potency (EC_50_ ~ 1 μM; SI > 150), the activity of polygodial (**1**) positions it as a potential alternative or adjunct therapy. Otherwise, the inactivity of 9-deoxymuzigadial (**2**) underscores the importance of specific structural features for antischistosomal activity, especially two geminal methyl groups at the C-4 position that can affect its metabolization and pharmacokinetics [15,16]. The contrasting results between polygodial (**1**) and 9-deoxymuzigadial (**2**) further support this hypothesis.

Building upon the in vitro findings, in silico analysis evaluated the drug-likeness of polygodial (**1**). Observing the bioavailability radar, it is noted that polygodial (**1**) shows properties comparable to those of the positive control, praziquantel, that includes data on liposolubility (LIPO), molecular flexibility (FLEX), number of unsaturation (UNSAT), solubility (SOLUB), polarity (POLAR), and molecular size (SIZE) [13]. Polygodial (**1**), falls within the radar, suggesting it to be a promising compound for progression to in vivo testing. As observed, polygodial (**1**) does not violate any of the drug-likeness rules established by Lipinski, Ghose, Veber, Egan, or Muegge. This compound displayed a log P_o/w_ < 5, a molecular weight (MW) < 400 Da, and a topological polar surface area (TPSA) < 140 Å, suggesting a high absorption and good oral availability [13]. Furthermore, no alerts for PAINS were detected for polygodial (**1**).

In vivo studies validated the antiparasitic potential of polygodial (**1**). A single oral dose of 400 mg/kg administered to *S. mansoni*-infected mice resulted in an approximately 44% reduction in worm burden. Notably, egg production was significantly suppressed, with reductions of around 70% in both fecal and intestinal samples. This finding is particularly important, as egg deposition is the primary driver of schistosomiasis-associated morbidity and transmission. In comparison, other natural products, such as dehydrodieugenol B [17] and the sesquiterpene nerolidol [18], have demonstrated more modest reductions in parasite burden and egg counts, further highlighting the superior efficacy of polygodial (**1**).

The exact mechanism by which polygodial (**1**) exerts its antiparasitic effects on schistosomes remains unclear. However, similar to other terpenes, its high hydrophobicity likely facilitates penetration across cellular membranes, enabling interactions with intracellular proteins and/or intra-organelle targets [19]. This property may contribute to its broad-spectrum activity and ability to disrupt critical biological processes in the parasite. The promising results obtained for polygodial (**1**) align with other studies that have highlighted the antiprotozoal and anthelmintic properties of natural drimane sesquiterpenes.

Despite these encouraging findings, additional studies are required to address some limitations. Detailed pharmacokinetic and toxicity assessments are essential to confirm the compound’s safety profile. Molecular similarity analyses using quantum mechanical descriptors could provide valuable insights into the compound’s biological activity [20,21]. Investigating its mechanism of action and exploring structural modifications could further enhance its therapeutic potential. Therefore, polygodial (**1**) demonstrates significant promise as a lead compound for schistosomiasis treatment, combining notable in vitro activity, high selectivity, and in vivo efficacy. These findings pave the way for the development of novel therapeutic agents to combat schistosomiasis, addressing a critical unmet need in global health.

## 4. Materials and Methods

### 4.1. General Procedures

NMR spectra were recorded using a Varian INOVA spectrometer (Santo André, SP, BR) operating at 500 and 125 MHz for ^1^H for ^13^C nuclei, respectively, using CDCl_3_ as the solvent and TMS as the internal standard. HR-ESIMS spectra were recorded using Bruker Daltonics MicroTOF QII spectrometer (Santo André, Brazil) operating in positive electron spray ionization mode. Silica gel (230−400 mesh, Merck, Darmstadt, Germany) was used for column chromatography procedures while silica gel 60 PF_254_ (Merck, Darmstadt, Germany) was employed for analytical TLC separations.

### 4.2. Plant Material

*D. brasiliensis* was collected on 3 December 2021, in Serra do Cipó National Park, Minas Gerais, Brazil. The species was identified by the botanist Dr. Guilherme M. Antar from Federal University of Espírito Santo, Brazil. The voucher specimen, registered as number 4105, was deposited at the herbarium of the University of São Paulo (SPF), Brazil.

### 4.3. Extraction

As previously reported [11], fresh branches of *D. brasiliensis* were dried at 30 °C and powdered to afford 316 g of plant material, which was extracted with hexane (10 × 500 mL) at room temperature. Combined extracts were concentrated under reduced pressure to afford 17 g of hexane extract.

### 4.4. Isolation of Polygodial (***1***) and 9-Deoxymuzigadial (***2***)

Part of the bioactive hexane extract from branches of *D. brasiliensis* (16 g) was chromatographed over silica gel eluted with increasing amounts of EtOAc in hexane (9:1; 8:2; 7:3; 6:4; 3:7) and pure EtOAc to afford five groups (A–E). After in vitro anti-*S. mansoni* evaluation, groups C and E displayed activity (100% mortality of the worms at 200 μg/mL). Group C was shown to be composed of 3,6-epidioxy-bisabola-1,10-diene, as previously reported [11]. Part of group E (200 mg) was subjected to further fractionation using silica gel soaked with AgNO_3_ eluted with hexane: Et_2_O 7:3 and 1:1 to give seven groups (E-1 to E-7). Polygodial (**1**) and 9-deoxymuzigadial (**2**) were isolated from groups E-3 (90 mg) and E-7 (22 mg), respectively.

*Polygodial* (**1**): White amorphous solid. HR-ESIMS: *m*/*z* 235.1679 [M + H]^+^ calculated for C_15_H_23_O_2_, 235.1698. ^1^H NMR (CDCl_3_, 500 MHz): δ 9.53 (d, *J* = 4.5 Hz, H-11), 9.45 (s, H-12), 7.14 (t, *J* = 2.8 Hz, H-7), 2.82 (br s, H-9), 2.49 (m, H-6_ax_), 2.33 (m, H-6_eq_), 1.82 (dd, *J* = 2.7 and 2.3 Hz, H-1_eq_), 1.50 (m, H-3_ax_ and H-2), 1.37 (td, *J* = 13.5 and 4.0 Hz, H-1_ax_), 1.28 (m, H-5) 1.24 (m, H-3), 0.96 (s, H-13), 0.94 (s, H-15), 0.93 (s, H-14). ^13^C NMR (CDCl_3_, 125 MHz): δ 202.0 (C-11), 193.2 (C-12), 154.2 (C-7), 138.2 (C-8), 60.3 (C-9), 48.9 (C-5), 41.7 (C-3), 39.5 (C-1), 36.8 (C-10), 33.1 (C-4), 33.0 (C-14), 25.2 (C-6), 21.9 (C-13), 18.0 (C-2), 15.2 (C-15).

*9-Deoxymuzigadial* (**2**): White amorphous solid. HR-ESIMS: *m*/*z* 233.1557 [M + H]^+^ calculated for C_15_H_21_O_2_ 233.1541. ^1^H NMR (CDCl_3_, 500 MHz): δ 9.53 (d, *J* = 4.2 Hz, H-11), 9.50 (s, H-12), 7.14 (m, H-7), 4.91 (br s, H-13_eq_), 4.72 (br s, H-13_ax_), 3.01 (br s, H-9), 2.44 (m, H-6), 2.11 (m, H-5), 2.02 (m, H-3), 1.91 (dt, *J* = 13.5 and 3.1 Hz, H-1_eq_), 1.70 (m, H-2_eq_), 1.62 (m, H-1_ax_), 1.08 (d, *J* = 6.5 Hz, H-14), 0.73 (s, H-15). ^13^C NMR (CDCl_3_, 125 MHz): δ 201.2 (C-11), 193.4 (C-12), 153.0 (C-7), 151.4 (C-4), 138.0 (C-8), 106.1 (C-13), 58.4 (C-9), 45.9 (C-5), 39.5 (C-1), 38.6 (C-3), 38.3 (C-10), 31.6 (C-2), 27.1 (C-6), 18.5 (C-14), 13.6 (C-15).

### 4.5. In Silico Analysis

In silico parameters were evaluated using the SwissADME platform developed and maintained by the Swiss Institute of Bioinformatics^®^, Lausanne, Switzerland [13]. On this website, 2D structural models of analyzed compounds were drawn in the molecular sketcher into ChemAxon’s Marvin JS window and transferred into a SMILES (simplified molecular-input line-entry system) format to predict suitable properties. Different pharmacokinetic parameters were assessed, including absorption, distribution, metabolism, and excretion (ADME). Additionally, drug-likeness criteria were analyzed based on the Lipinski (Pfizer, Hong Kong), Veber (GlaxoSmithKline, Hong Kong), and Muegge (Bayer, Leverkusen, Germany) filters. The identification of pan-assay interference compounds (PAINS) was also included in the evaluation.

### 4.6. Animals, Parasites, and Cells

The *Schistosoma mansoni* life cycle (BH strain) was maintained at Guarulhos University (Guarulhos, SP, Brazil), utilizing *Biomphalaria glabrata* snails as intermediate hosts and Swiss mice as definitive hosts. Both snails and mice were kept in a controlled environment at 25 °C with 50% relative humidity, a 12-h light/dark cycle, and free access to food and water. Swiss mice (four weeks old) were subcutaneously infected with *S. mansoni* cercariae harvested from infected snails.

Vero cells (monkey kidney epithelial cells; ATCC CCL-81) were sourced from the American Type Culture Collection (Manassas, VA, USA). The cells were cultured in Dulbecco’s Modified Eagle Medium (DMEM) supplemented with 2 mM L-glutamine, 10% heat-inactivated fetal bovine serum, and antibiotics (100 U/mL penicillin and 100 µg/mL streptomycin). Cultures were maintained in 25 cm^2^ flasks (Corning, Tewksbury, MA, USA) at 37 °C in a humidified incubator with 5% CO_2_. Subculturing was performed using a 0.25% trypsin-EDTA solution [22].

### 4.7. In Vitro Antiparasitic Assay

Adult *S. mansoni* worms were harvested from infected mice via dissection 49 days post-infection and maintained in RPMI 1640 medium supplemented with 5% fetal calf serum, 100 U/mL penicillin, and 100 µg/mL streptomycin. Test compounds **1** and **2** were prepared in RPMI medium at an initial concentration of 50 µM and placed into 24-well plates (Corning, New York, NY, USA). Six concentrations were tested in a serial dilution format over 72 h at 37 °C in a 5% CO_2_ atmosphere. Each well contained one male and one female worm. Controls included 0.5% DMSO as the negative control and praziquantel as the positive control [23].

Worm viability was assessed under an inverted microscope at intervals of 0, 24, 48, and 72 h. Mortality was defined as the complete absence of movement observed for at least one minute [24]. All experiments were performed in triplicate, and results were expressed as the percentage of surviving worms compared to the controls.

### 4.8. In Vitro Cytotoxicity Assay

Cytotoxicity was evaluated using the MTT assay. Vero cells were seeded at a density of 2 × 10^3^ cells per well in 96-well plates and exposed to serial dilutions of sesquiterpenes, starting at a concentration of 200 µM. Six concentrations were tested in a serial dilution format over 72 h at 37 °C in a 5% CO_2_ atmosphere [23,25]. After the incubation period, MTT solution was added, and the plates were incubated for an additional 4 h. Absorbance was measured at 595 nm using an Epoch spectrophotometer (BioTek Instruments, Winooski, VT, USA).

Cell viability was calculated as a percentage relative to untreated controls. The selectivity index (SI) was determined as the ratio of the compound’s CC_50_ in Vero cells to its EC_50_ against *S. mansoni*. Each experiment was conducted in triplicate to ensure reproducibility [26].

### 4.9. In Vivo Efficacy in Mice Infected with S. mansoni

The in vivo efficacy of compound **1** was tested using a murine model of schistosomiasis. Three-week-old Swiss mice were subcutaneously infected with 80 cercariae of *S. mansoni* per animal [27]. On day 42 post-infection, the mice were divided into three groups (*n* = 5 per group) and treated via oral gavage with a single dose of compound **1** (400 mg/kg), praziquantel (400 mg/kg) or vehicle (water).

Mice were euthanized on day 56 post-infection using CO_2_ inhalation. The worms were then recovered, sexed (separated into male and female), and counted to assess worm burden reduction. Egg burden reduction was evaluated using the oogram method for intestinal eggs and the Kato–Katz technique for fecal eggs. To minimize bias, all analyses were performed by investigators blinded to the treatment groups [28].

### 4.10. Data Analysis

Statistical analyses were conducted using GraphPad Prism 8.0 (San Diego, CA, USA). EC_50_ and CC_50_ values were calculated from sigmoidal dose–response curves. Comparisons of worm burden and egg counts between control and treatment groups were performed using the non-parametric Kruskal–Wallis test. A *p*-value of less than 0.05 was considered statistically significant [23].

## 5. Conclusions

In conclusion, the results of the present study demonstrated the in vitro and in vivo antischistosomal activities of chemically related drimane sesquiterpene polygodial (**1**) and 9-deoxymuzigadial (**2**), both isolated from the hexane extracts from branches of *D. brasiliensis* (Winteraceae). In vitro assays showed compound **1** worm mortality with an EC_50_ value of 12.5 μM, whereas **2** showed to be inactive. Additionally, both **1** and **2** were not cytotoxic to mammalian cells and displayed good absorption and oral bioavailability when evaluated in silico. Based on these results, the effects of polygodial (**1**) in vivo using a murine infection model were evaluated, where there was a reduction in worm burden (44.09%) and in egg production in feces (71.80%) and intestines (69.49%), indicating a similar efficacy to the positive control praziquantel. Therefore, polygodial represents a promising candidate for the development of new drugs for the treatment of schistosomiasis.

## Figures and Tables

**Table 1:** Antischistosomal activity of natural sesquiterpenes polygodial (**1**) and 9-deoxymuzigadial (**2**) compared to the positive control praziquantel.

Compound	*S. mansoni* EC_50_/μM		Vero EC_50_/μM	SI	
	Male	Female		Male	Female
polygodial (**1**)	9.6 ± 0.5	9.3 ± 1.7	>200	>21	>22
9-deoxymuzigadial (**2**)	NA	NA	>200	-	-
praziquantel	1.1 ± 0.7	1.3 ± 0.8	>200	>182	>154

EC_50_: effective concentration 50%; NA: not active; SI: Selective Index.

**Table 2:** In silico and ADME properties of polygodial (**1**) compared to the positive control praziquantel.

Parameters	Polygodial (1)	Praziquantel
Molecular Weight (Da)	234.33	312.41
TPSA (Å)	34.14	40.62
log P_o/w_	3.23	3.00
log S	−3.20	−3.52
Gastrointestinal absorption	High	High
Lipinksi	Yes; 0 violation	Yes; 0 violation
Ghose	Yes	Yes
Veber	Yes	Yes
Egan	Yes	Yes
Muegge	Yes	Yes
Bioavaliability Score	0.55	0.55
PAINS	0 alert	0 alert

## Data Availability

The data that support the findings of this study are available from the corresponding author upon reasonable request.

## Supplementary Materials

The following supporting information can be downloaded at: https://www.mdpi.com/article/10.3390/molecules30020267/s1, Figure S1: ESI-HRMS of polygodial (**1**), Figure S2: ^1^H NMR spectrum of polygodial (**1—**δ, CDCl_3_, 500 MHz); Figure S3: ^13^C NMR spectrum of polygodial (**1—**δ, CDCl_3_, 125 MHz); Figure S4: ESI-HRMS of 9-deoxymuzigadial (**2**); Figure S5: ^1^H NMR spectrum of 9-deoxymuzigadial (**2—**δ, CDCl_3_, 500 MHz); Figure S6: ^13^C NMR spectrum of 9-deoxymuzigadial (**2—**δ, CDCl_3_, 125 MHz); Table S1: Molecular structure and assignment data for compounds **1** and **2** respectively.
