# Supplementary material for: In Vitro and In Vivo Evaluation of the Antischistosomal Activity of Polygodial and 9-Deoxymuzigadial Isolated from Drimys brasiliensis Branches
Source: Molecules. 2025 Jan 11;30(2):267. doi: 10.3390/molecules30020267 (PMC11767830; doi:10.3390/molecules30020267)
Supplement: Supplementary file 1 [file molecules-30-00267-s001.zip › molecules-3372162-supplementary.pdf]

In vitro and *in vivo* antischistosomal activity of polygodial and 9-deoxymuzigadial isolated from *Drimys brasiliensis* branches

Eric Umehara[1], Rayssa A. Cajas[2], Gabriel B. Conceição[2], Guilherme M. Antar[3], Adriano D. Andricopulo[4,5], Josué de Moraes[2,5,6], and João Henrique G. Lago[1,5]

SUPPLEMENTARY MATERIAL

[1]Centro de Ciências Naturais e Humanas, Universidade Federal do ABC, Santo André 09280-560, SP, Brazil;

[2]Núcleo de Pesquisas em Doenças Negligenciadas, Universidade Guarulhos, Guarulhos 07023-070, SP, Brazil;

[3]Departamento de Ciências Agrárias e Biológicas, Universidade Federal do Espírito Santo, São Mateus, 29932540, ES, Brazil;

[4]Laboratório de Química Medicinal e Computacional, Centro de Pesquisa e Inovação em Biodiversidade e Fármacos, Instituto de Física de São Carlos, Universidade de São Paulo, São Carlos 13563-120, SP, Brazil;

[5]Centro de Pesquisa e Inovação Especial em Ciências da Descoberta de Medicamentos (CEPIMED), Universidade de São Paulo, São Carlos 13563-120, SP, Brazil;

[6]Núcleo de Pesquisas em Doenças Negligenciadas, Instituto Científico e Tecnológico, Universidade Brasil, São Paulo 08230-030, SP, Brazil;

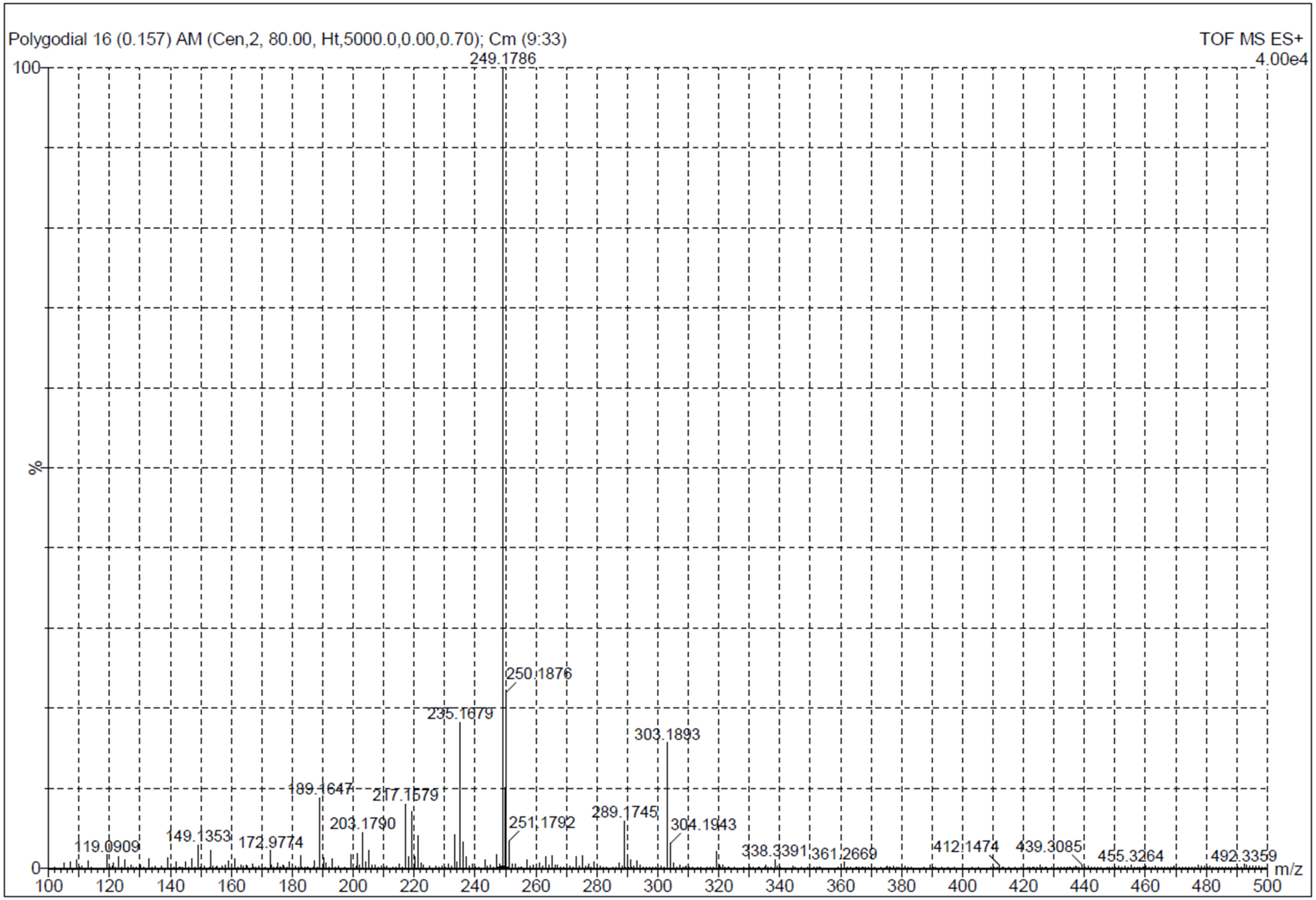

Figure S1. ESI-HRMS of polygodial (**1**).

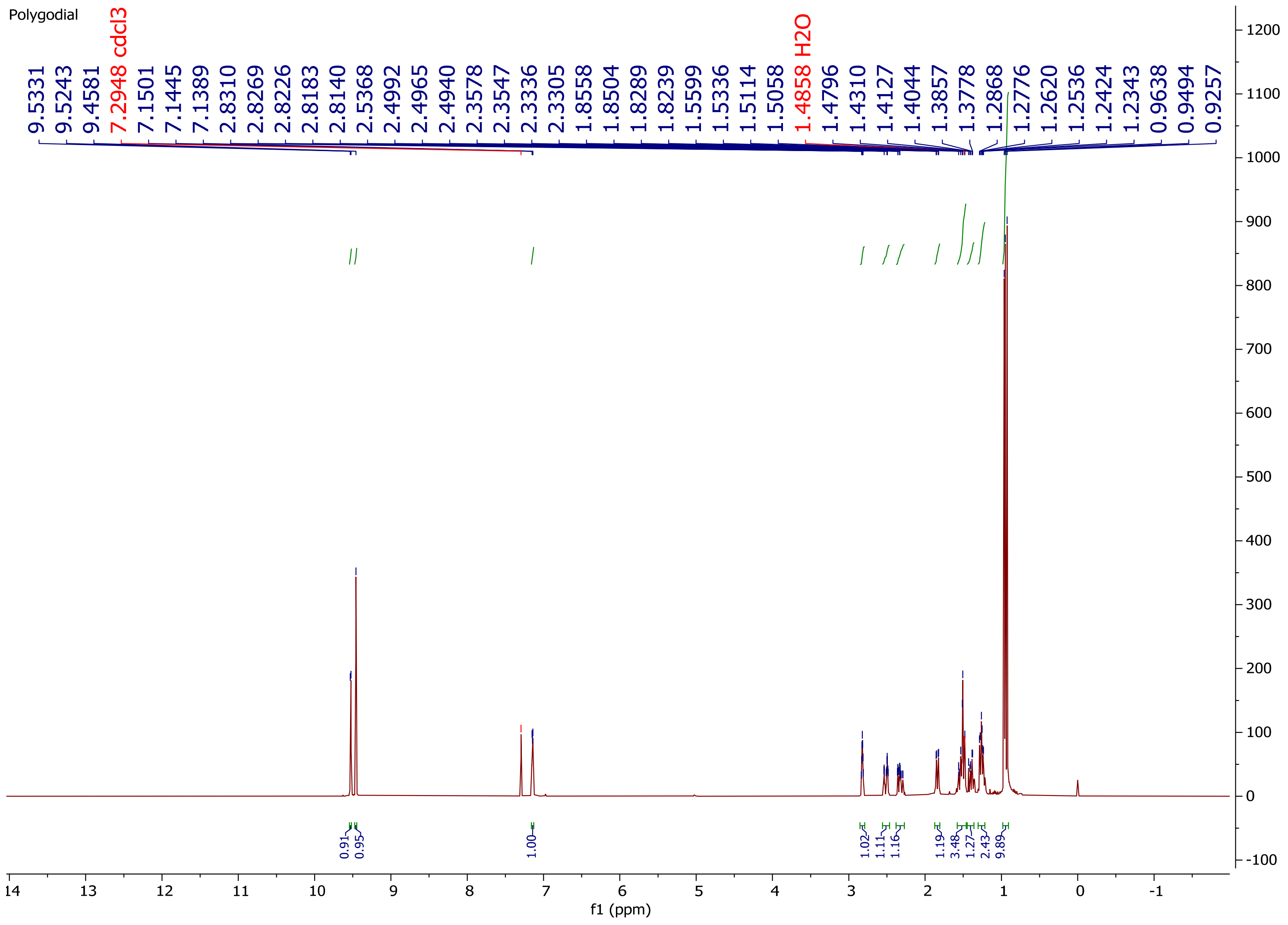

Figure S2. ^{1}H NMR spectrum of polygodial (**1** – δ, $CDCl_3$, 500 MHz).

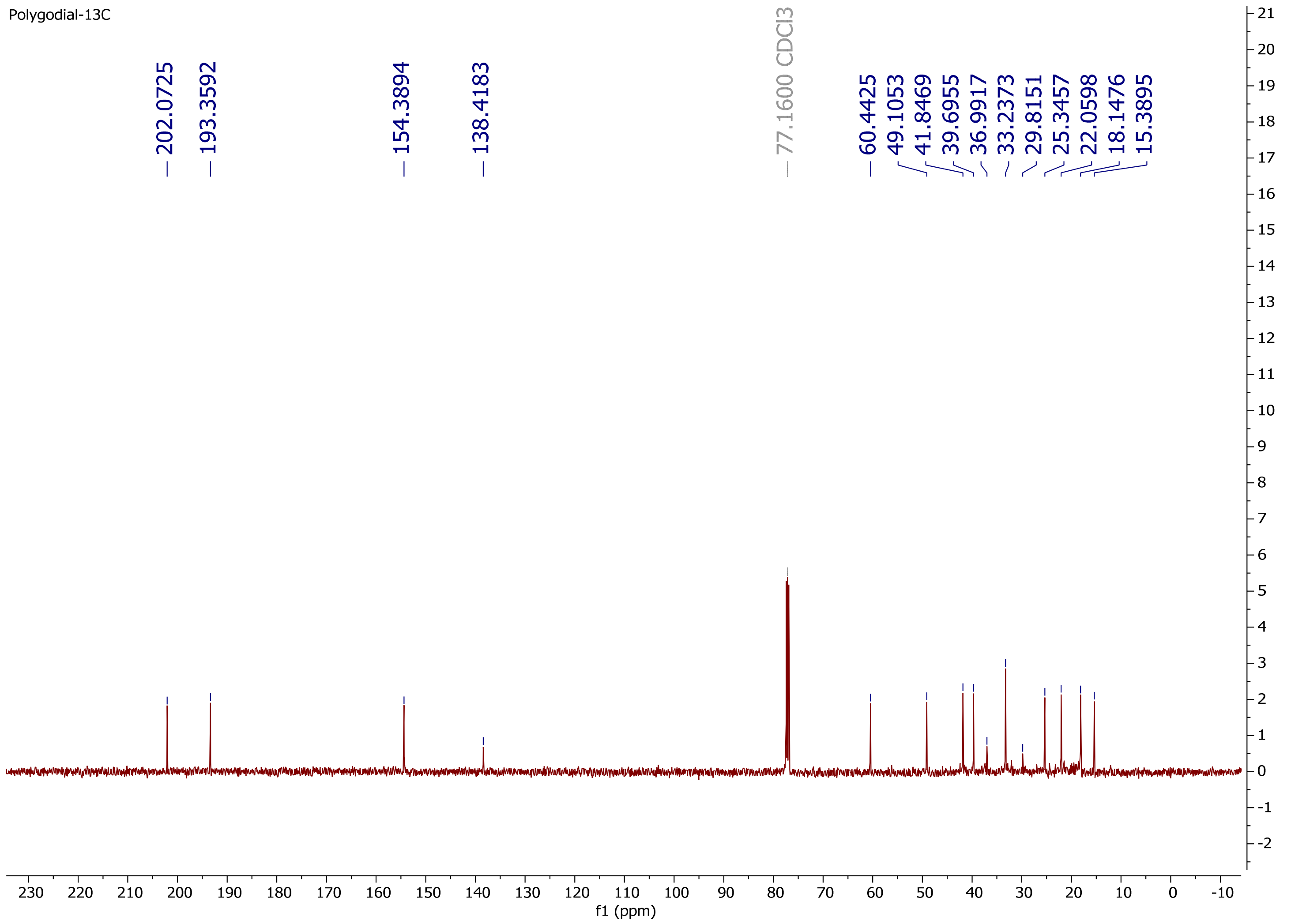

Figure S3. ^{13}C NMR spectrum of polygodial (**1** – δ, $CDCl_3$, 125 MHz).

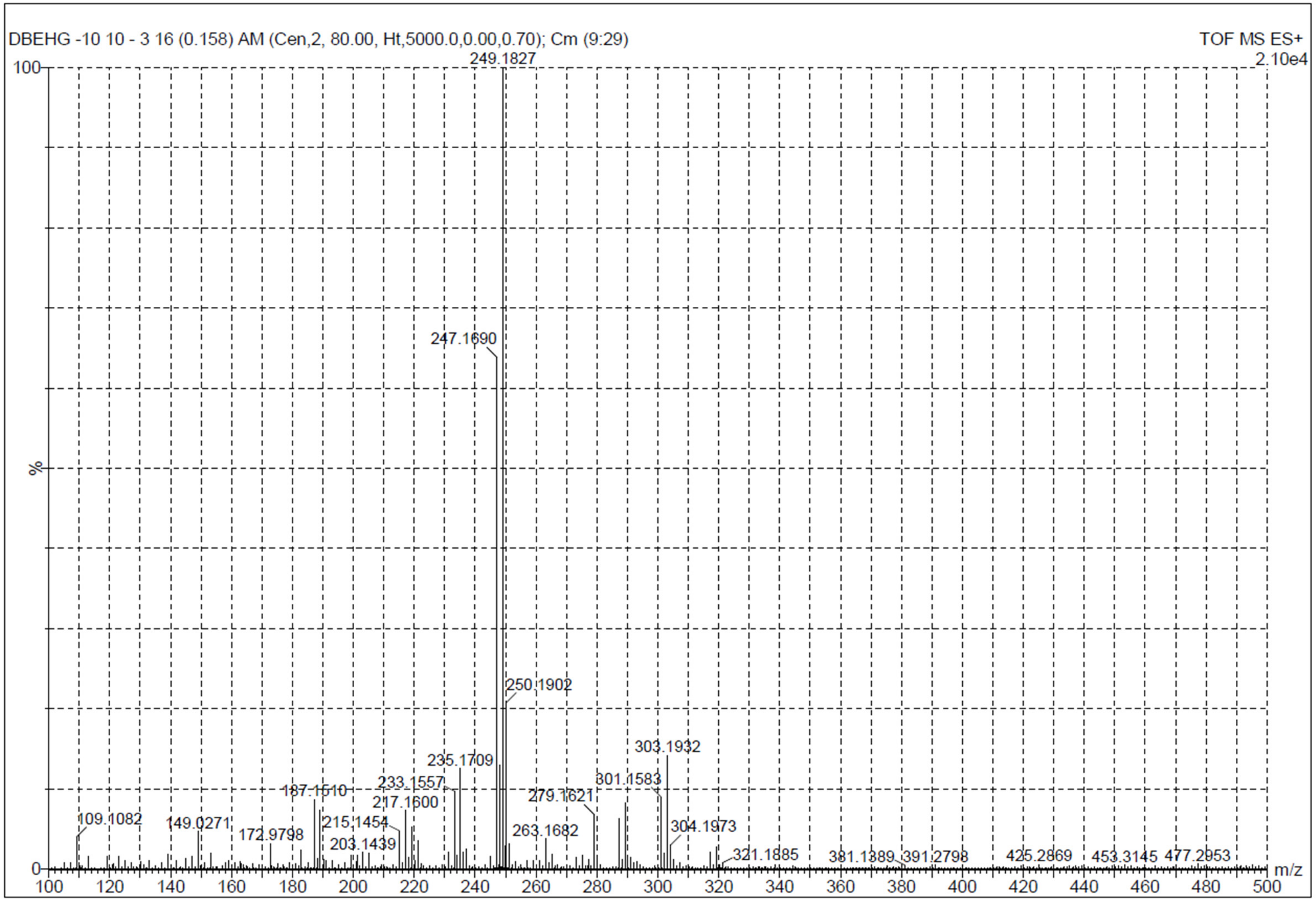

Figure S4. ESI-HRMS of 9-deoxymuzigadial (**2**).

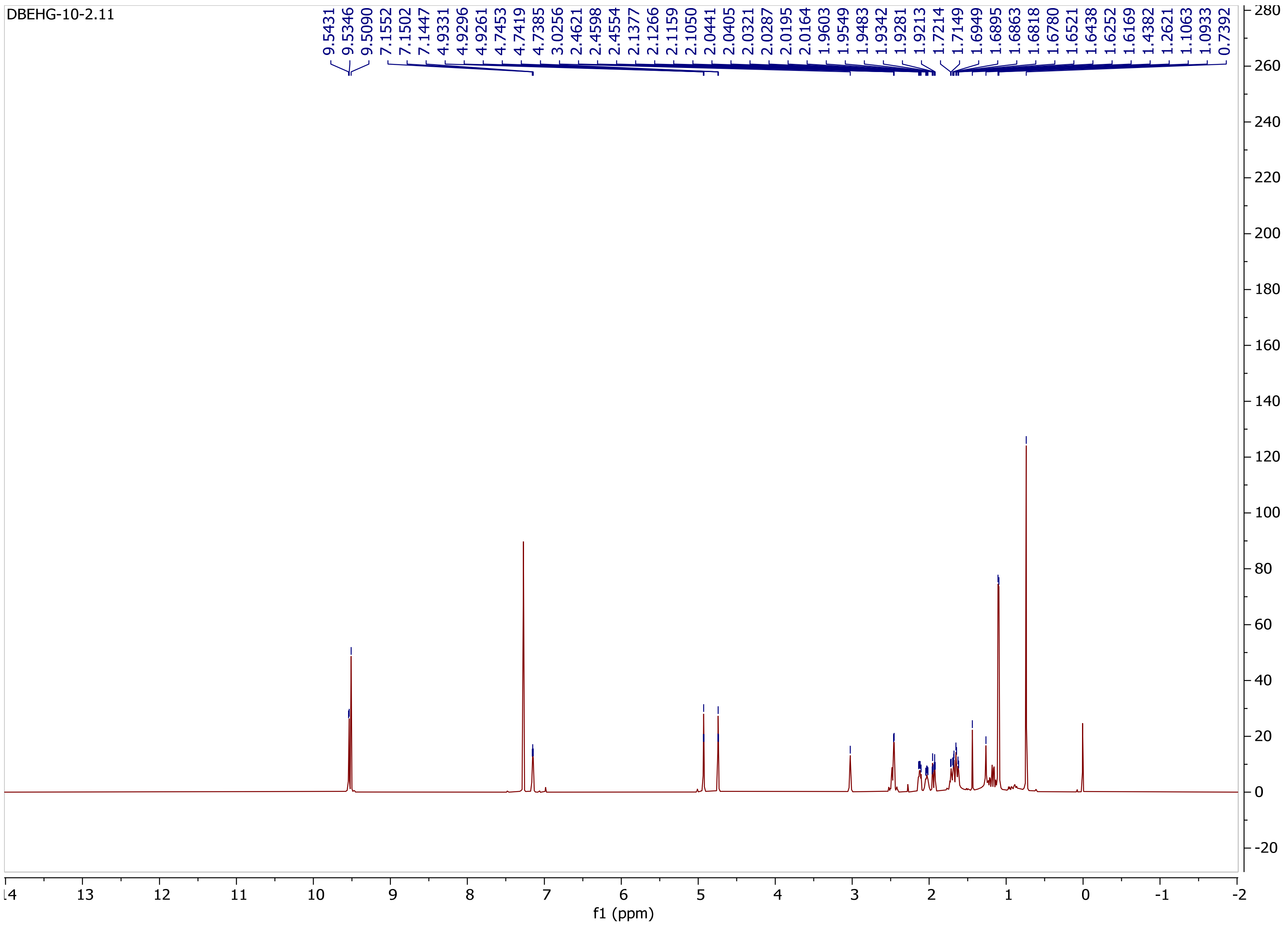

Figure S5. ^{1}H NMR spectrum of 9-deoxymuzigadial (**2** – δ, $CDCl_3$, 500 MHz).

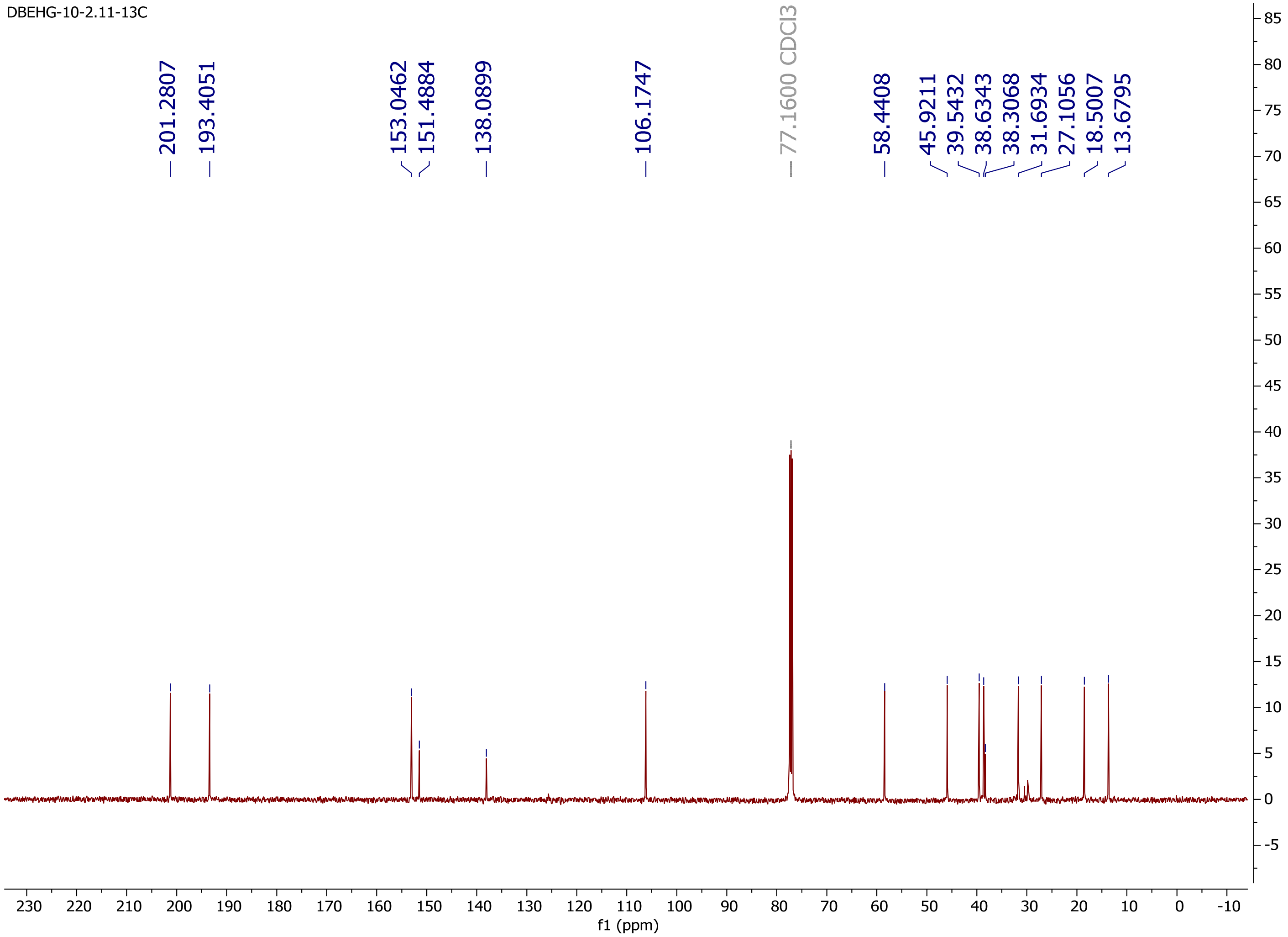

Figure S6. ^{13}C NMR spectrum of 9-deoxymuzigadial (**2** – δ, $CDCl_3$, 125 MHz).

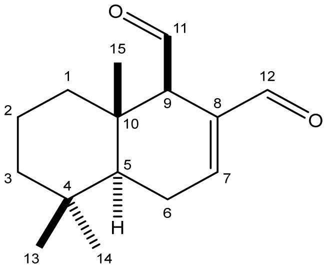

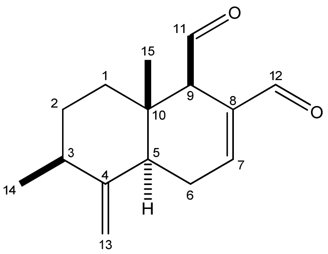

	Compound 1		Compound 2	
**#**	**δH / ppm**	**δC / ppm**	**δH / ppm**	**δC / ppm**
**1**	eq 1.82 (dd, *J* = 2.7 and 2.3 Hz)<br>ax 1.37 (td, *J* = 13.5 and 4.0 Hz)	39.5	eq 1.91 (dt, *J* = 13.5 and 3.1 Hz)<br>ax 1.62 (m)	39.5
**2**	1.50 (m)	18.0	eq 1.70 (m)<br>ax 1.15 (dd, *J* = 12.8 and 3.7 Hz)	31.6
**3**	ax 1.50 (m)<br>eq 1.24 (m)	41.7	2.02 (m)	38.6
**4**	-	33.1	-	151.4
**5**	1.28 (m)	48.9	2.11 (m)	45.9
**6**	eq 2.33 (m)<br>ax 2.49 (m)	25.2	2.44 (m)	27.1
**7**	7.14 (t, *J* = 2.8 Hz)	154.2	7.14 (m)	153.0
**8**	-	138.2	-	138.0
**9**	2.82 (br s)	60.3	3.01 (br s)	58.4
**10**	-	36.8	-	38.3
**11**	9.53 (d, *J* = 4.5 Hz)	202.0	9.53 (d, *J* = 4.2 Hz)	201.2
**12**	9.45 (s)	193.2	9.50 (s)	193.4
**13**	0.96 (s)	21.9	eq 4.91 (br s)<br>ax 4.72 (br s)	106.1
**14**	0.93 (s)	33.1	1.08 (d, *J* = 6.5 Hz)	18.5
**15**	0.94 (s)	15.2	0.73 (s)	13.6

Table S1. Molecular structure and assignment data for compounds **1** and **2** respectively.
